# ?Description of a new species of the genus *Neopseustis* Meyrick, 1909 from China, with a new classification of the genus (Lepidoptera, Neopseustoidea, Neopseustidae)

**DOI:** 10.3897/zookeys.1078.75461

**Published:** 2021-12-15

**Authors:** Siyao Huang, Yongxiang Hou, Lijuan Zhu, Yongqiang Xu, Min Wang, Xiaoling Fan, Yang Long, Wa Da, Liusheng Chen

**Affiliations:** 1 Department of Entomology, College of Plant Protection, South China Agricultural University, Guangzhou 510642, Guangdong, China South China Agricultural University Guangzhou China; 2 Tibet Plateau Institute of Biology, Lhasa 850001, Xizang Autonomous Prefecture, China Tibet Plateau Institute of Biology Lhasa China; 3 Guangdong Academy of Forestry, Guangzhou 510520, Guangdong, China Guangdong Academy of Forestry Guangzhou China

**Keywords:** Classification, Himalaya, India, *Neopseustina*, new species, Sichuan, Xizang

## Abstract

A new species of the genus *Neopseustis* Meyrick, 1909, *Neopseustischentangensis* S.Y. Huang & Chen **sp. nov.**, which was confirmed by both morphological and molecular methods, is described from Xizang, China. This is currently the westernmost species in Asia of the primitive lepidopteran family Neopseustidae. The new species is externally reminiscent of *N.moxiensis* Chen & Owada, 2009; however, it can be easily distinguished from the latter by comparison of the male genitalia and is further distinguished by the large genetic distance in DNA barcodes (COI). The adult and genitalia of the new and similar species have been illustrated. Utilizing our new data, a new classification of the genus is provided, with its members subdivided into four species groups: the *meyricki*-group, the *moxiensis*-group, the *bicornuta*-group, and the *chentangensis*-group, which are supported by both molecular and morphological evidence. A checklist of the genus and a key to the species groups are also provided.

## ?Introduction

The family Neopseustidae is a small and archaic lepidopteran family known only by four genera and 14 species and with a peculiar disjunct distribution. Ten of these species are found in Southeast Asia, and the rest are found in South America (Davis 1975; Davis and Nielsen 1980; Davis and Nielsen 1985; Liao et al. 2021). Kristensen (1999) listed several probable autapomorphies for the family, mainly taken from the head, thorax, and abdomen, including the facial scales being restricted to paired lateral and usually swollen patches, the prominent apodemal plate invaginated from the upper base of the propecoxal bridge, the male sternum VII with medial spinose process, etc. Faucheux et al. (2006) studied the antennal flagellum sensilla of several neopseustid species and stated that one sensillum type, called “multiporous large sensillum basiconicum” in their work, is unknown in other lepidopterans except Neopseustidae; thus, the presence of such a sensillum constitutes an autapomorphy of the family. Recent molecular studies have brought new knowledge concerning the phylogenetic position of the family. Mutanen et al. (2010) and Regier et al. (2013) recovered the clade Acanthopteroctetidae+Neopseustidae, but with weak support. Kristensen et al. (2015) found that with the discovery of Acanthopteroctetidae, the clade Acanthopteroctetidae+Acanthopteroctetidae+Neopseustidae (abbreviated as the AAeN clade in that work) was strongly supported, and Acanthopteroctetidae was found to be sister to Neopseustidae. The close relationship is supported by the sharing of a strong precoxal bridge between the prothoracic pleuron and sternum. Moreover, the AAeN clade was found to be sister to all the Heteroneura (Kristensen et al. 2015). Regier et al. (2015) also reported the grouping of Neopseustidae and Acanthopteroctetidae, and this clade was found to be supported by the presence of the smooth intercalary sclerotization and the alignment of antennal scale sockets in longitudinal rows in the antenna. Regier et al. (2015) also suggested that the former monotypic Neopseustoidea should include also the Acanthopteroctetidae and Acanthopteroctetidae, and that together they form the sister group to Heteroneura. Externally, Neopseustidae adults are small to medium-sized moths with long antennae and semitransparent, thin-scaled wings, and they resemble some families in the order Neuroptera. Little is known about the biology of Neopseustidae. Adults can be active during the day or night, flying above bushes or attracted to light traps (Liao et al. 2021; present study). As for the immature stages, Grehan (1991) suggested that the disjunct distribution of the plant family Lardizabalaceae fitted well with that of the family Neopseustidae, but no feeding had ever been recorded. Regier et al. (2015) reported an astonishing parasitoid immature stage on Limacodidae of *Neopseustismeyricki* Hering, 1925, but later the larvae of this Taiwanese species have been found to feed on Ampelopsisbrevipedunculatavar.hancei, family Vitaceae (DearLep 2021), suggesting that the former record was based on an error. For other Neopseustidae there is no information on immatures.

To date, two genera and seven species have been recorded from mainland China, which are distributed in Henan, Sichuan, Guizhou, Hunan, Guangxi, and Guangdong provinces (Davis 1975; Chen et al. 2009; Liao et al. 2021). Xizang Autonomous Prefecture, also known as Tibet, is a biological hotspot region located in southwestern China and is well known for its various biotopes. Due to the diverse vegetation types found at different altitudes, this area is home to many families of Lepidoptera, and new discoveries are frequently reported. Neopseustidae are currently unknown for the Xizang fauna. During a survey conducted in May 2021, the first author unexpectedly captured a strange looking individual of this intriguing family from Chentang Town, Xigaze City, located in southern Xizang. After careful examination, this individual has been proven to be an unknown species, which is described herein. This is currently westernmost distribution record of the genus *Neopseustis* in Asia, and it is also the first record of the family in Xizang. Furthermore, we provide a new classification for the species in the genus *Neopseustis*, based both on molecular and morphological evidence.

## ?Materials and methods

### ?Morphological study.

Specimens examined were collected during daytime, using an insect net, or with a light trap at night and subsequently deposited in the collection of the South China Agricultural University (SCAU), Guangzhou. Photographs of the adult and the habitat of the new species were taken using a Sony DSC-RX100 v. 1.00 camera. The abdomens were removed and macerated in 10% NaOH for about 2 min at about 95 °C for dissection of the genitalia. The genitalia were removed from the abdomen and mounted in glycerin for photographing. Photographs of the genitalia of the new species were taken under a Keyence VHX-5000 digital microscope, and those of other taxa were taken under a Zeiss SteReo Discovery V.12 digital microscope. Photographs of adults and genitalia were processed using Adobe Photoshop CS5 software. The terminology for adults and genitalia follows Davis (1975) and Liao et al. (2021).

### ?Molecular analysis.

Our molecular analysis comprised 19 samples, six of which are newly obtained COI sequences for DNA barcoding. Detailed information on these samples is provided in Table 1. Three COI sequences of three species of the genus *Apoplania* Davis, 1975, two sequences of the monobasic genus *Synempora* Davis & Nielsen, 1980, and two sequences of one species of the genus *Neopseustis* were downloaded from BOLDSystem (www.boldsystems.org). Five sequences belonging to three species of the genus *Neopseustis* and a sequence of *Endoclitadavidi* (Poujade, 1886), which was used as the outgroup in our phylogenetic analysis, were downloaded from NCBI (www.ncbi.nlm.nih.gov). The details of protocols for DNA extraction, amplification, and sequencing have been provided in previous publications (Fan et al. 2016; Tang et al. 2017; Huang et al. 2019). The sequences were aligned using Clustal W (Thompson et al. 1997) implemented in MEGA v. 7.0 (Kumar et al. 2016) with default parameters, and genetic distances were calculated using Kimura-2-parameter models implied by the same software. Maximum likelihood analyses were performed using IQ-tree v. 2.1.3 (Minh et al. 2020) with the branch support values evaluated by 1000 ultrafast bootstrap (UFBS) replicates (Minh et al. 2013) on the web server (http://iqtree.cibiv.univie.ac.at/). We considered the branch support strong when the UFBS was 95 or higher. Genetic distances were calculated using the Kimura-2-parameter models implied by the same software. All sequences were submitted to GenBank under the submission numbers OK148463 to OK148468. The specimens with voucher numbers CT1, BX1, YJ1, YJ2, MX1, and SZ1 were deposited in SCAU.

**Table 1.** Voucher information and GenBank accession numbers for COI sequences of the Neopseustidae specimens and outgroup in this study. Newly obtained sequences are indicated by an asterisk (*).

Taxon	Locality	Date	Voucher Number	Accession Number
*Neopseustischentangensis* S.Y. Huang & Chen sp. nov.	Xizang, China	V.2021	CT1	OK148463*
*Neopseustisrectagnatha* Liao, Chen & Huang, 2021	Hunan, China	VIII.2020	HAUHL039474	MW804623
*Neopseustisrectagnatha* Liao, Chen & Huang, 2021	Hunan, China	VIII.2020	HAUHL039473	MW804622
*Neopseustisrectagnatha* Liao, Chen & Huang, 2021	Hunan, China	VI. 2020	HAUHL040282	MW804609
*Neopseustisarchiphenax* Meyrick, 1928	Henan, China	VII. 2002	LNAUT030–14	N/A
*Neopseustisarchiphenax* Meyrick, 1928	Henan, China	VII. 2002	LNAUT031–14	N/A
*Neopseustissinensis* Davis, 1975	Sichuan, China	VII. 2009	BX1	OK148464*
*Neopseustissinensis* Davis, 1975	Sichuan, China	VII. 2009	YJ1	OK148465*
*Neopseustismeyricki* Hering, 1925	Taiwan, China	N/A	LS-06–0068	GU828566
*Neopseustismoxiensis* Chen & Owada, 2009	Sichuan, China	VIII. 2004	MX1	OK148466*
*Neopseustisfanjingshana* Yang, 1988	Hunan, China	VIII. 2019	HAUHL041880	MW804624
*Neopseustisfanjingshana* Yang, 1988	Hunan, China	VIII. 2008	SZ1	OK148467*
*Neopseustisbicornuta* Davis, 1975	Sichuan, China	VII. 2009	YJ2	OK148468*
*Apoplaniavaldiviana* Davis & Nielsen, 1985	Cautin, Chile	XII. 1982	LNAUT029–14	N/A
*Apoplaniapenai* Davis & Nielsen, 1980	Chiloe Island, Chile	XII. 1981	LNAUT022–14	N/A
*Apoplaniachilensis* Davis, 1975	Curico Las Tablas, Chile	II. 1985	LNAUT019–14	N/A
*Synemporaandesae* Davis & Nielsen, 1980	Sagrario Puerto, Argentina	II. 1979	LNAUT041–14	N/A
*Synemporaandesae* Davis & Nielsen, 1980	Aguas Calientes, Argentina	II. 1979	LNAUT042–14	N/A
*Endoclitadavidi* (Poujade, 1886)	Hunan, China	XI. 2015	HN20170409020	KY928030

## ?Taxonomy

### ?Genus Neopseustis

Taxon classificationAnimaliaLepidopteraNeopseustidae

Meyrick, 1909

7F4981CB-3C35-5EBB-886A-4111C099129E

Neopseustis Meyrick, 1909: 436.

#### Type species.

*Neopseustiscalliglauca* Meyrick, 1909, by monotypy. [Type locality: Khasi Hills, Assam, India].

### ? Neopseustis chentangensis

Taxon classificationAnimaliaLepidopteraNeopseustidae

S.Y. Huang & Chen sp. nov.

BE572CC1-D94C-559E-B1C3-649688E15502

http://zoobank.org/9E16636E-F0EE-4738-9DD9-4A6259EB96B6

Figures 1 , 3–10

#### Type material.

***Holotype***: male, altitude 2600 m, 23.V.2021, Chentang Town, Dingjie County, Xigaze City, Xizang Autonomous Prefecture, P.R. China, leg. Siyao Huang, voucher number and dissection number CT1 (SCAU).

#### Diagnosis.

Externally, *N.chentangensis* resembles *N.moxiensis* Chen & Owada, 2009 (Fig. 2, 11–12) from Moxi, western Sichuan, share a fuscous ground colour on both wings. However, the new species can be immediately distinguished from *N.moxiensis* by the combination of the following characters: smaller size (length of forewing 8.7 mm vs 9 mm in holotype of *N.moxiensis*), narrower forewing (slightly broader in *N.moxiensis*), patches along forewing costa slenderer and darker (patches along forewing costa thicker and lighter in *N.moxiensis*), narrower hindwing and light fuscous ground colour (broader hindwing and light yellowish brown ground colour in *N.moxiensis*), and more uniform fringe in both wings (cilia clearly chequered, especially in hindwing in *N.moxiensis*). In the male genitalia, *N.chentangensis* can be easily distinguished from *N.moxiensis* by the shape of the latero-posterior process of anellus, which is long, robust, and L-shaped; the distal end is deeply bifurcated and forms two sharp processes bending anteriorly (in *N.moxiensis*, the latero-posterior process of anellus is not L-shaped and bent anteriorly at the tip.). The tegumenal lobe is significantly slenderer after it is flattened (in *N.moxiensis* the tegumenal lobe is much broader when it is flattened), the valvae lack the uncinate process apically, and long and thick processes ventrally (both processes present in *N.moxiensis*). The anterior arms of the vinculum are more slender (these arms are broader and shorter in *N.moxiensis*). From the other congeners, *N.chentangensis* can be simply distinguished by the shape of its latero-posterior process of anellus mentioned above.

#### Description.

**Adult**: length of forewing 8.7 mm. Antennae brownish dorsally. Head, thorax, and abdomen uniformly brownish. Forewing nearly oval, apex slightly pointed. Forewing ground color pale yellowish fuscous, with four fuscous patches along costa to apex. Several irregular black or brownish transverse lines present in the median and submarginal zones. A row of brownish spots extending from apex to anal angle along termen. Fringe fuscous from apex to anal angle, slightly checkered with creamy white in dorsum. Hindwing oval, ground color uniformly light fuscous. Hindwing apex with light yellowish spot at the marginal zone. Fringe generally fuscous from apex to anal angle and slightly checkered with creamy white around anal angle.

**Figures 1, 2.**
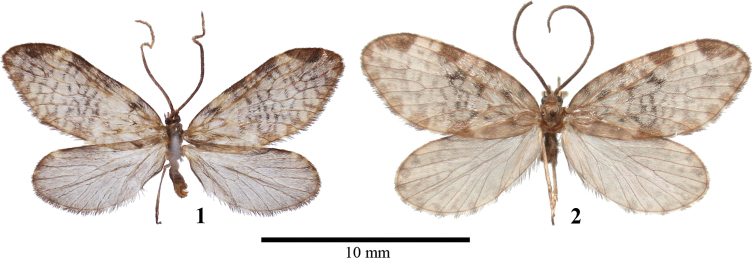
Males of *Neopseustis* spp. **1***Neopseustischentangensis* S.Y. Huang & Chen sp. nov., holotype, Chentang, Xizang, CT1 **2***N.moxiensis*, holotype, Moxi, Sichuan, MX1.

**Figures 3–12.**
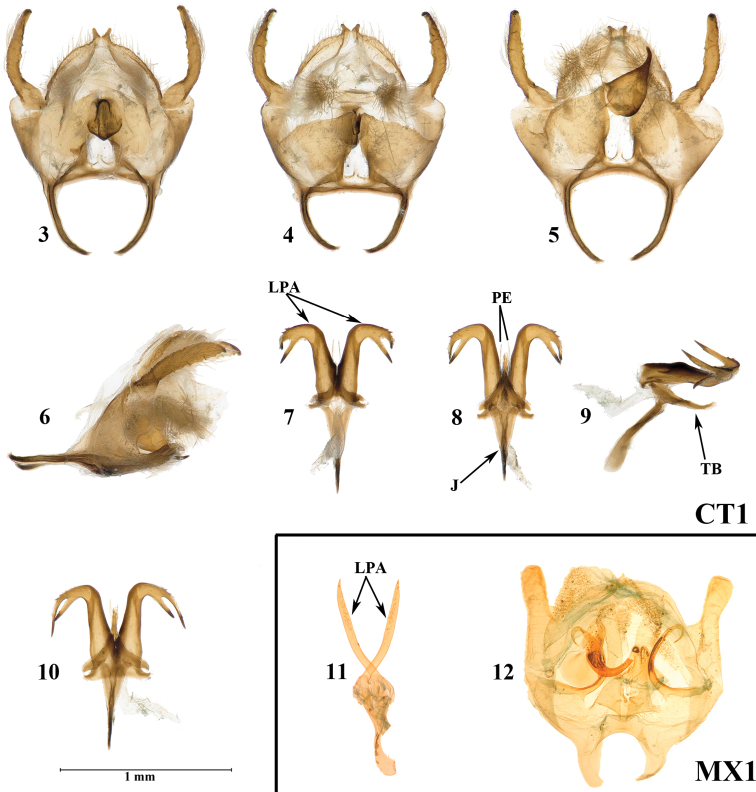
Male genitalia of *Neopseustis* spp. **3–10***Neopseustischentangensis* sp. nov., holotype, dissection number CT1 **3** genitalia capsule in natural shape with anellus-juxta-parameres removed, dorsal view **4** same, in ventral view **5** genitalia capsule flattened with anellus-juxta-parameres removed **6** genitalia capsule in natural shape with anellus-juxta-parameres removed, in lateral view **7** anellus-juxta-parameres in natural shape, in dorsal view **8** same, in ventral view **9** same, in lateral view **10** anellus-juxta-parameres flattened, in ventral view **11, 12***Neopseustismoxiensis*, holotype, dissection number MX1 **11** anellus-juxta-parameres flattened, in ventral view **12** genitalia capsule flattened with anellus-juxta-parameres removed. J = Juxta; LPA = lateroposterior process of anellus; PE = parameres; TB = transverse bar. Scale bar: 1 mm (Figures **3–10**).

**Male genitalia**: Uncus fused with tegumen, bifurcate basally and forming two short and distally rounded lobes. Gnathos strongly sclerotized thoroughly, consisting of a medially curved, short, and robust distal process and a large and thick base. Socii rounded, densely setose. Tegumenal lobe slightly curved outwards beyond the base and gradually narrowing towards its tip. Valvae totally fused with vinculum, broad and nearly trapezoid in natural shape. Vinculum broad posteriorly, abruptly narrowing anteriorly and forming long and slender arms. Lateroposterior process of anellus generally L-shaped, thick, and robust, with the tip deeply bifurcate and forming two sharp processes bending anteriorly. Two denticles present at the upper margin of dorsal process. Paired processes of anellus absent. Transverse bar in lateral view obtuse-triangular and slightly bending upwards near tip, while in dorsal and ventral views generally triangular with the lower angles shallowly bifurcate. Juxta in lateral view slightly curved outwards and nearly broad Y-shaped in dorsal and ventral views. Parameres short and setose-like, weakly sclerotized, situated between the two lateroposterior processes of anellus.

**Female.** Unknown at present.

#### Bionomics.

**Figures 13, 14.**
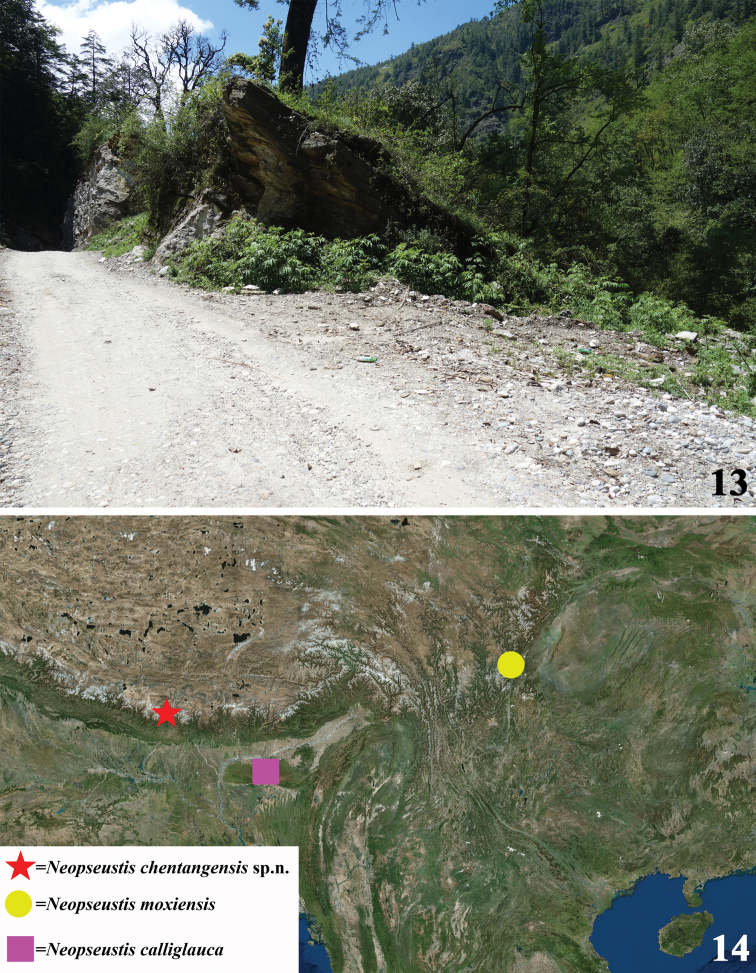
**13** collecting site of *Neopseustischentangensis* in Chentang Town, Xizang **14** Distribution map of some *Neopseustis* spp. in Asia.

The holotype of *N.chentangensis* was spotted weakly flying above bushes during the daytime at an altitude about 2600 m. The collecting site (Fig. 13) is located at the edge of a forest along a road in a valley.

#### Distribution.

Currently only known from the type locality, Chentang Town (Fig. 14).

#### Etymology.

The specific epithet chentangensis is derived from the type locality, Chentang Town.

#### Molecular analysis.

**Figure 15.**
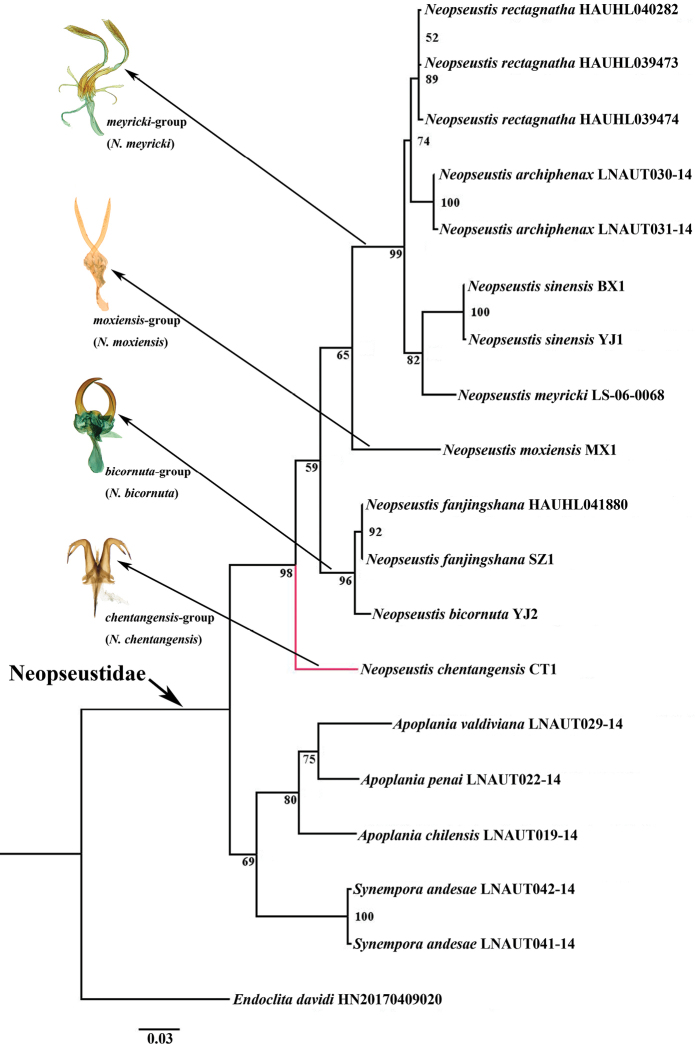
Phylogenetic tree of *Neopseustidae* based on an analysis of the COI barcoding region, using the maximum likelihood method. The genus *Neopseustis* is divided into four groups with their corresponding anellus-juxta-parameres illustrated on the left side.

The Kimura-2-parameter distance of the genus *Neopseustis*, based on COI barcoding, is given in Table S1. The maximum interspecific divergence occurred between *N.chentangensis* and *N.moxiensis*, which was 11.7%, and the minimum interspecific divergence occurred between *N.fanjingshana* and *N.bicornuta*, which was 1.5%. According to the table, *N.chentangensis* is genetically distinct from its congeners, with the genetic divergence varying from 7.6 to 11.5%. Based on the ML tree (Fig. 15) constructed using the COI barcoding region, the genus *Neopseustis* was monophyletic (UFBS = 98), and subsequently diverged into four clades, with three of them receiving strong support (UFBS > 95). *Neopseustischentangensis* was found to be sister to all the remaining taxa in the current study.

## ?Discussion

Although Davis (1975) and Liao et al. (2021) considered that the genus *Neopseustis* should be subdivided into two groups based on the morphology of male genitalia and molecular phylogenetic analysis, we consider that this genus may actually comprise of at least four groups, after utilizing more data from previously unsampled taxa. The first group, as already recognized by Davis (1975) and Liao et al. (2021), consists of *N.rectagnatha* Liao, Chen & G.H. Huang, 2021; *N.meyricki* Hering, 1925; *N.archiphenax* Meyrick, 1928; and *N.sinensis* Davis, 1975, and is called the *meyricki*-group. The second group consists only of *N.moxiensis* Chen & Owada, 2009, and is called the *moxiensis*-group. The third group, consisting of *N.fanjingshana* Yang, 1988 and *N.bicornuta* Davis, 1975, is called the *bicornuta*-group and probably also includes the unsampled type species, *N.calliglauca* Meyrick, 1909, based on the morphology of its anellus-juxta-parameres. The fourth group consists of only *N.chentangensis* S.Y. Huang & Chen and is called the *chentangensis*-group. Among these four groups, except for the *meyricki*-group which is unique in having well-developed parameres and a narrow, short, and forked lateroposterior process on the anellus, the *moxiensis*, *bicornuta*, and *chentangensis* groups all share ill-developed parameres, but they can be distinguished from each other by the combination of features in the male genitalia. The *moxiensis*-group is characterized by the latero-posterior process of the anellus covered by dense spinules from middle to distal end, in addition to the valvae which have an uncinate process apically and a long and thick process ventrally. The *bicornuta*-group is characterized by the latero-posterior process of the anellus smooth from middle to distal end and the absence of ventral process in the valvae. The *chentangensis*-group is characterized by latero-posterior process of anellus long, L-shaped with apex deeply bifurcating and bending anteriorly, and gnathos with a large and thick base.

It is rather intriguing that although *N.chentangensis* is similar externally only to *N.moxiensis* in *Neopseustis*, among the whole genus, they have the greatest genetic divergence. Their male genitalia structures are also considerably different from each other, suggesting that the relationship between them is distant. We believe that their external similarity may probably due to their parallel evolution under similar environments. Unlike their relatively whitish congeners inhabiting the mid- and lower-elevation mountainous areas in India, mainland China, and Taiwan, these two species all inhabit high mountainous areas above 2500 m, and the similar cool climate in high elevation areas in western Sichuan and southern Xizang. This probably may have led to the evolution of their dark wing coloration which can help them absorb heat faster. This assumption is also supported by the studies of Wu et al. (2019), Trullas et al. (2007), and Pereboom and Biesmeijer (2003), who produced similar conclusions.

The former westernmost record of the genus *Neopseustis* is the type species *N.calliglauca*, which is found in the Khasi Hills in India. The current record of this new species is situated about 520 km northwest of Khasi Hill, and thus is currently the westernmost record of the genus. The discovery of *N.chentangensis* in Chentang, on the southern slope of the Himalayas in Xizang, suggests that the investigation of the microlepidopteran fauna is still inadequate in remote areas along the Himalaya. The collection site of the new species is very close to the border of China and Nepal, and Neopseustidae are unknown in Nepal. It can be expected that this species or other new species will someday be discovered in Nepal or Bhutan. Moreover, Neopseustidae are also expected in the southeastern part of Xizang, where no species are currently found. It is possible that the absence of this family there is only due to a lack of surveys, as poor transportation conditions in past decades makes this paradise of moths difficult to access.

### ?Checklist of the genus *Neopseustis* Meyrick, 1909

***meyricki* -group**

*N.archiphenax* Meyrick, 1928

Distribution. Myanmar, China (Sichuan, Henan)

*N.meyricki* Hering, 1925

Distribution. China (Taiwan)

*N.rectagnatha* Liao, Chen & G.H. Huang, 2021

Distribution. China (Hunan, Guangxi, Guangdong)

*N.sinensis* Davis, 1975

Distribution. China (Hunan, Sichuan)

***moxiensis* -group**

*N.moxiensis* Chen & Owada, 2009

Distribution. China (Sichuan)

***bicornuta* -group**

*N.bicornuta* Davis, 1975

Distribution. China (Sichuan)

*N.calliglauca* Meyrick, 1909

Distribution. India (Khasi Hills, Meghalaya)

*N.fanjingshana* Yang, 1988

Distribution. China (Guizhou, Hunan)

***chentangensis* -group**

*N.chentangensis* S.Y. Huang & Chen, sp. nov.

Distribution. China (Xizang)

### ?Key to the species-groups of the genus *Neopseustis* based on male genitalia structures

**Table:** 

1	Parameres well developed and narrow; lateroposterior process of anellus short, and forked	***Neopseustismeyricki* -group**
–	Parameres poorly developed	**2**
2	Latero-posterior process of anellus long, L-shaped with apex deeply bifurcate, bending anteriorly	***Neopseustischentangensis* -group**
–	Latero-posterior process of anellus apex not bifurcate and pointed posteriorly	**3**
3	Latero-posterior process of the anellus covered by dense spinules from middle to distal end; valvae with an uncinate process apically and a long and thick process ventrally	***Neopseustismoxiensis* -group**
–	Latero-posterior process of the anellus smooth from middle to distal end; valvae without a ventral process	***Neopseustisbicornuta*-group**

## Supplementary Material

XML Treatment for Neopseustis

XML Treatment for Neopseustis chentangensis

## Supplementary materials

Supplementary material 1 Table S1. The Kimura-2-parameter distance on COI sequences between different taxon of the genus *Neopseustis* sampled for the current study Data type molecular data https://binary.pensoft.net/file/623627This dataset is made available under the Open Database License (http://opendatacommons.org/licenses/odbl/1.0/). The Open Database License (ODbL) is a license agreement intended to allow users to freely share, modify, and use this Dataset while maintaining this same freedom for others, provided that the original source and author(s) are credited.Siyao Huang
