# Supplementary material for: ?Description of a new species of the genus Neopseustis Meyrick, 1909 from China, with a new classification of the genus (Lepidoptera, Neopseustoidea, Neopseustidae)
Source: Zookeys. 2021 Dec 15;1078:35–48. doi: 10.3897/zookeys.1078.75461 (PMC8695565; doi:10.3897/zookeys.1078.75461)
Supplement: Supplementary material 1 — Table S1. The Kimura-2-parameter distance on COI sequences between different taxon of the genus Neopseustis sampled for the current study [file zookeys-1078-035-s001.docx]

Table S1. The Kimura-2-parameter distance on COI sequences between different taxon of the genus *Neopseustis* sampled for the current study*.*

|  | Samples | 1 | 2 | 3 | 4 | 5 | 6 | 7 | 8 | 9 | 10 | 11 | 12 | 13 |
| --- | --- | --- | --- | --- | --- | --- | --- | --- | --- | --- | --- | --- | --- | --- |
| 1 | N_chentangensis_CT1 | 0.000 |  |  |  |  |  |  |  |  |  |  |  |  |
| 2 | N_rectagnatha*_*HAUHL040282 | 0.088 | 0.000 |  |  |  |  |  |  |  |  |  |  |  |
| 3 | N_rectagnatha*_*HAUHL039473 | 0.088 | 0.003 | 0.000 |  |  |  |  |  |  |  |  |  |  |
| 4 | N_rectagnatha*_*HAUHL039474 | 0.086 | 0.005 | 0.005 | 0.000 |  |  |  |  |  |  |  |  |  |
| 5 | N_archiphenax_LNAUT030-14 | 0.095 | 0.023 | 0.023 | 0.023 | 0.000 |  |  |  |  |  |  |  |  |
| 6 | N_archiphenax_LNAUT031-14 | 0.099 | 0.027 | 0.027 | 0.026 | 0.003 | 0.000 |  |  |  |  |  |  |  |
| 7 | N_sinensis_BX1 | 0.093 | 0.051 | 0.051 | 0.052 | 0.056 | 0.059 | 0.000 |  |  |  |  |  |  |
| 8 | N_sinensis_YJ1 | 0.095 | 0.052 | 0.052 | 0.054 | 0.057 | 0.061 | 0.002 | 0.000 |  |  |  |  |  |
| 9 | N_meyricki_LS-06-0068 | 0.093 | 0.043 | 0.043 | 0.044 | 0.054 | 0.058 | 0.049 | 0.051 | 0.000 |  |  |  |  |
| 10 | N_moxiensis_MX1 | 0.117 | 0.090 | 0.090 | 0.088 | 0.095 | 0.099 | 0.099 | 0.101 | 0.102 | 0.000 |  |  |  |
| 11 | N_fanjingshana_HAUHL041880 | 0.076 | 0.084 | 0.084 | 0.083 | 0.090 | 0.093 | 0.097 | 0.099 | 0.102 | 0.085 | 0.000 |  |  |
| 12 | N_fanjingshana_SZ1 | 0.076 | 0.085 | 0.085 | 0.083 | 0.090 | 0.093 | 0.097 | 0.099 | 0.102 | 0.085 | 0.000 | 0.000 |  |
| 13 | N_bicornuta_YJ2 | 0.079 | 0.090 | 0.090 | 0.088 | 0.095 | 0.099 | 0.099 | 0.100 | 0.102 | 0.090 | 0.015 | 0.015 | 0.000 |
